# L-Dopa Modulation of Brain Connectivity in Parkinson’s Disease Patients: A Pilot EEG-fMRI Study

**DOI:** 10.3389/fnins.2019.00611

**Published:** 2019-06-14

**Authors:** Stefania Evangelisti, Francesca Pittau, Claudia Testa, Giovanni Rizzo, Laura Ludovica Gramegna, Lorenzo Ferri, Ana Coito, Pietro Cortelli, Giovanna Calandra-Buonaura, Fabio Bisquoli, Claudio Bianchini, David Neil Manners, Lia Talozzi, Caterina Tonon, Raffaele Lodi, Paolo Tinuper

**Affiliations:** ^1^Functional MR Unit, Department of Biomedical and NeuroMotor Sciences, University of Bologna, Bologna, Italy; ^2^EEG and Epilepsy Unit, Geneva University Hospitals, Geneva, Switzerland; ^3^Department of Physics and Astronomy, University of Bologna, Bologna, Italy; ^4^Department of Biomedical and NeuroMotor Sciences, University of Bologna, Bologna, Italy; ^5^IRCCS Istituto delle Scienze Neurologiche di Bologna, Bologna, Italy; ^6^Functional Brain Mapping Lab, Department of Fundamental Neurosciences, University of Geneva, Geneva, Switzerland

**Keywords:** Parkinson’s disease, EEG-fMRI, functional connectivity, L-dopa, pilot study

## Abstract

Studies of functional neurosurgery and electroencephalography in Parkinson’s disease have demonstrated abnormally synchronous activity between basal ganglia and motor cortex. Functional neuroimaging studies investigated brain dysfunction during motor task or resting state and primarily have shown altered patterns of activation and connectivity for motor areas. L-dopa administration relatively normalized these functional alterations. The aim of this pilot study was to examine the effects of L-dopa administration on functional connectivity in early-stage PD, as revealed by simultaneous recording of functional magnetic resonance imaging (fMRI) and electroencephalographic (EEG) data. Six patients with diagnosis of probable PD underwent EEG-fMRI acquisitions (1.5 T MR scanner and 64-channel cap) before and immediately after the intake of L-dopa. Regions of interest in the primary motor and sensorimotor regions were used for resting state fMRI analysis. From the EEG data, weighted partial directed coherence was computed in the inverse space after the removal of gradient and cardioballistic artifacts. fMRI results showed that the intake of L-dopa increased functional connectivity within the sensorimotor network, and between motor areas and both attention and default mode networks. EEG connectivity among regions of the motor network did not change significantly, while regions of the default mode network showed a strong tendency to increase their outflow toward the rest of the brain. This pilot study provided a first insight into the potentiality of simultaneous EEG-fMRI acquisitions in PD patients, showing for both techniques the analogous direction of increased connectivity after L-dopa intake, mainly involving motor, dorsal attention and default mode networks.

## Introduction

Alterations in the temporal pattern of neuronal discharge have been found to be associated with parkinsonian symptoms (Marsden and Obeso, 1994; Obeso et al., 1997), but the central mechanisms underlying motor deficits in Parkinson’s Disease (PD) is still unclear (Brown and Marsden, 1998; Jankovic, 2008).

Findings based on functional neurosurgery in PD patients suggest the existence of excessive neuronal synchronization mainly in the subthalamic nucleus (STN) and globus pallidus (Levy et al., 2002b; Williams et al., 2002; Brown, 2003), in particular within the beta band (13–30 Hz). It has been demonstrated that treatment with L-dopa not only reduces this synchronization, but it is also associated with a stronger synchronization within the gamma band (30–100 Hz) (Brown et al., 2001; Levy et al., 2002a,b; Priori et al., 2002; Williams et al., 2002; Brown, 2003). Moreover, abnormal synchronized activity in the basal ganglia may be coupled to activity in the motor cortex: oscillatory synchronization within and between cortical areas is increasingly recognized as a key mechanism in motor organization (Ohara et al., 2001; Serrien and Brown, 2003) and over the last few decades, electroencephalographic (EEG) studies have shown a high incidence of background and focal intermittent EEG slowing in PD (Yeager et al., 1966; McPherson, 1970). The correlation between motor disability and slowing of the background EEG suggests that this effect may be related to failure of nigrostriatal modulation of basal ganglia inputs to the cortex (Neufeld et al., 1988). In PD patients, relevant changes may also occur in the pattern of synchronization across distributed areas of the cortex (cortico–cortical coherence) (Silberstein et al., 2005; Hirschmann et al., 2011, 2013). This finding is especially significant in the beta and gamma bands, given the importance of cortico–cortical coherence at these frequencies in motor organization and the evidence that basal ganglia activity is preferentially synchronized in these bands. In particular, it has been shown that, when exploring resting state scalp EEG, there is a correlation between EEG–EEG coherence over the 10–35 Hz range and the severity of parkinsonism, and that the reduction of cortical coupling correlates with L-dopa therapy, STN stimulation and with the consequent clinical improvement (Silberstein et al., 2005). The frequency-specific functional connectivity between basal ganglia and cortex was investigated in PD patients also using simultaneous magnetoencephalography (MEG) and local field potentials recordings (Hirschmann et al., 2011, 2013). Coherent activity was observed in the beta range in the ipsilateral sensorimotor and premotor cortices, as well as in the alpha range in the ipsilateral temporal lobe, and, besides, it was found that beta coherence between primary motor cortex (M1) and STN was suppressed after the administration of L-dopa.

Brain magnetic resonance techniques are valuable methods to explore the pathophysiological bases of PD for both structural and functional aspects, and might provide new potential biomarkers for the *in vivo* differential diagnosis with atypical parkinsonism (Bajaj et al., 2017; Heim et al., 2017; Zanigni et al., 2017; Morisi et al., 2018). In particular, functional neuroimaging techniques have been mostly applied to give further insights into the pathophysiology of motor dysfunction in PD and to explore brain dysfunction underlying core motor symptoms such as bradykinesia and rigidity by using specific motor tasks during the MRI acquisitions (Rizzo et al., 2012). Overall, these studies mainly reported altered functional activation in supplementary motor area (SMA), pre-SMA, M1, premotor cortex, prefrontal cortex, parietal cortex, cingulum, basal ganglia, thalamus and cerebellum. Some discrepancies in the findings could be related to the stage of disease and/or to differences between the motor fMRI paradigms. L-dopa administration relatively normalized the functional activation patterns (Haslinger et al., 2001; Buhmann et al., 2003; Wu and Hallett, 2005). More recently, resting state fMRI has been used to investigate brain functional connectivity in PD patients. Typically, such patients show lower functional connectivity, compared to healthy subjects, for M1, SMA, dorso-lateral prefrontal cortex, temporal lobe, putamen, substantia nigra, striatum, STN, thalamus, cerebellum and the default mode network, and alterations in connectivity were likewise appreciably normalized by L-dopa administration (Wu et al., 2009, 2011, 2012; Sharman et al., 2013). The differences among the results reported by resting state fMRI studies might depend, in addition to clinical samples and methodological differences, on resting state being an uncontrolled condition, with a risk of unstable wakefulness (Tagliazucchi and Laufs, 2014).

The combined acquisition of EEG and fMRI was originally developed with a clinical interest to investigate the neuronal activity and the hemodynamic response simultaneously to try to detect and localize epileptic discharges (Rosenkranz and Lemieux, 2010; Huster et al., 2012; Vitali et al., 2015). In the recent years, however, EEG-fMRI became a promising techniques also in experimental neuroscience, in psychiatric and behavioral disorders related to mesocorticolimbic dopamine pathway dysfunction, such as attention-deficit hyperactivity disorder or schizophrenia, in narcolepsy, and in neurodegenerative disorders, such as Alzheimer’s disease (Boecker et al., 2014; Lei et al., 2014; Schneider et al., 2015; Drissi et al., 2016; Brueggen et al., 2017; Syed Nasser et al., 2019).

Given the evidence that both fMRI and EEG independently provided important insights into PD pathophysiology and specific functional alterations, the aim of this exploratory pilot study was to investigate the effect of acute L-dopa administration on functional connectivity in early-stage PD patients as revealed by simultaneous recording of hemodynamic (fMRI) and electrical (EEG) activity. The combined use of EEG and fMRI techniques guaranteed a controlled resting state acquisition and might provide a more comprehensive perspective on functional connectivity changes in PD: the simultaneous acquisition of these techniques gives the possibility to have both high temporal resolution EEG electrical measure and fMRI whole-brain neurometabolic evaluation with good spatial resolution at the same time and in the same group of patients.

## Materials and Methods

### Subjects

Eight PD patients were recruited at the Movement Disorders outpatients center, IRCCS Istituto delle Scienze Neurologiche di Bologna, DIBINEM, University of Bologna, Italy. Inclusion criteria were: diagnosis of probable idiopathic PD, according to Gelb’s criteria (Gelb et al., 1999); ongoing L-dopa therapy at stable dosage in at least two previous months (other drug therapies, such as dopaminergic agents, MAO or COMT inhibitors, were stopped during the week before the EEG-fMRI session); L-dopa efficacy documented by kinetic-dynamic monitoring within 1 year from the session. Exclusion criteria were: cognitive impairment and/or inability to provide informed consent; MR contraindications; neuroimaging findings inconsistent with PD diagnosis, severe involuntary movements; antidepressant or other therapies acting on central nervous system.

The same neurologist (GR) performed neurological evaluation of all patients using the Unified Parkinson’s Disease Rating Scale (UPDRS) – III testing and the Hoehn and Yahr scale (HY) (Hoehn and Yahr, 1967).

This study was carried out in accordance with the Declaration of Helsinki. The protocol was approved by the Local Public Health Service (AUSL) of Bologna Ethics Committee and all participants provided written informed consent. No healthy controls were included, as the primary goal of the study was to compare connectivity patterns before and after the administration of L-dopa in PD patients.

### EEG-fMRI Acquisitions

Each participant was instructed to go to bed at his or her preferred time on the evening before the examination, to fast after midnight, and not to take the first daily dose of L-dopa tablet (100 mg) in the morning. All acquisitions were performed between 8 am and 1 pm. Patients first underwent UPDRS-III and HY testing before taking L-dopa (i.e., in the OFF state), then the MR compatible EEG cap was set-up. This was followed by the positioning in the MR scanner and the OFF state acquisition. Immediately afterward, L-dopa was administered and patients underwent UPDRS-III retesting every 10 min in order to identify the ON state (i.e., under L-dopa effect). Within 15 min following the onset of the effect of the medication, patients were repositioned in the MR scanner and the ON state EEG-fMRI was acquired. Scalp EEG was recorded with an MR-compatible 64-channel cap (Brain Vision), according to the 10–20 International System (reference: central top electrode, near Cz). The recording resolution was 0.1 μV and the sampling frequency was 5 kHz. The ECG trace was continuously recorded at the same sampling frequency by means of a specific ECG cable.

Brain MRI acquisitions were performed using a 1.5 T scanner (GE Medical Systems Signa HDx 15) equipped with an 8-channel phased array brain coil. Participants were instructed to stay during the scan awake, relaxed and motionless with their eyes closed, and to avoid goal-directed attention. Simultaneously to the EEG recordings, two resting state runs were acquired during the OFF and the ON conditions (13 min for each condition) with a pure axial GE-EPI sequence (Gradient Echo–Echo Planar Imaging, TR/TE = 3000/40 ms, flip angle = 90°, FOV = 24 cm, voxel = 1.875 × 1.875 × 4 mm); for each run the first five volumes were discarded. A high-resolution volumetric sequence was acquired at the end of the session (T1-weighted Fast SPoiled Gradient, TR/TE/TI = 12/5/600 ms, FOV = 25.6 cm, voxel = 1 mm^3^).

### Data Analysis

Subjects’ UPDRS rating was compared between OFF and ON states with the Wilcoxon test.

#### EEG

Electroencephalographic signal preprocessing was performed with Brain Vision Analyzer 2.0 software. This included gradient artifact off-line correction and EEG signal filtering (Allen et al., 2000). A 50-Hz low-pass filter was also applied to remove the remaining artifact. The ballistocardiogram and eye-movements and blinking artifacts were removed by Independent Components Analysis (ICA) (Bénar et al., 2003). Finally, signals were down-sampled to 250 Hz and examined by visual inspection to remove sections containing muscular artifacts and sleep patterns. In both conditions, the artifact-free sections were concatenated.

To perform EEG-directed connectivity analysis, a previously published approach (Coito et al., 2015, 2016) was used, and is summarized below. For regional electrical source imaging (ESI), the forward model consisted of a simplified realistic head model [Locally Spherical Model with Anatomical Constraints, LSMAC (Birot et al., 2014)]. Based on the MNI template, about 5000 solution points were equally distributed within gray matter, which represented the solution space. A linear distributed inverse solution with biophysical constraints was then used to calculate the 3D current density distribution [Local Auto-Regressive Averages, LAURA (Grave de Peralta Menendez et al., 2004)]. The brain was then parcellated into 82 regions of interest (ROIs) using the AAL atlas, and the source activity of the solution point closest to the geometric center of each ROI was considered as the representative source activity of the ROI. To account for the changing tridimensional orientation of the source dipoles, these were projected at each time point on the predominant dipole direction of each ROI to obtain scalar values of the current density. Directed functional connectivity was determined with a Granger-causality measure: the time-varying weighted Partial Directed Coherence (wPDC) (Astolfi et al., 2008; Van Mierlo et al., 2011; Plomp et al., 2014). This gave a 4-dimensional matrix (82 ROIs × 82 ROIs × time × frequency) that was then reduced to the theta, alpha and beta frequency bands and averaged over time. Specifically, The time–frequency distribution of the power spectral density (PSD) was calculated using the S-transform (ST). To determine the PSD for each voxel in the inverse space, ST was computed for each scalp electrode, and source estimation was then applied to this frequency-domain complex data. The mean PSD for each patient was computed and normalized (0–1) across regions, time, and frequencies (1–100 Hz) by subtracting the minimum power and dividing by the range. The PDC (partial directed coherence) was analyzed for each frequency band. Time-varying PDC estimates directed interactions between pairs of signals in the time and frequency domain using adaptive multivariate autoregressive models (AMVAR). We estimated the time-varying AMVAR parameters by means of a recursive least squares (RLS) algorithm (Astolfi et al., 2008). PDC values were scaled, in the same way as the ST, and multiplied by the spectral power (weighted PDC, wPDC) (Van Mierlo et al., 2011; Plomp et al., 2014). The summed outflow for a given ROI and time point was defined as the sum of wPDC values from that ROI to all others ROIs at that time point. For each time point, the summed outflow for a given ROI and time point was then computed, as the sum of wPDC values from that ROI to all others. For each patient, the summed outflow of each region at each time point of the OFF segments was compared to each time point of the ON segments with a non-parametric test (Mann–Whitney–Wilcoxon, *p* < 0.05). As 82 different regions are tested at each band, a Bonferroni multiple comparisons correction is considered. EEG connectivity analysis and ESI were carried out using the software Cartool^1^ and Matlab 2012b (MathWorks Inc.).

#### fMRI

Functional magnetic resonance imaging data processing and analysis were mainly performed using FSL (version 4.1.4) (Jenkinson et al., 2012). Functional connectivity was investigated with a seed-based approach, using four distinct ROIs: right and left supplementary motor area (R-SMA, L-SMA), and right and left precentral gyri (R-PG, L-PG). Seeds were drawn in the MNI space as spheres of radius 5 mm centered at the following coordinates: R-SMA (6, 0, 54), L-SMA (-6, 0, 54), R-PG (44, -8, 38), and L-PG (-44, -8, 38).

The measured ECG trace was used to remove cardiac physiological noise from fMRI data by means of a retrospective method [RETROICOR, RETROspective Image-based CORrection (Glover et al., 2000)]. We used the RETROICOR implementation in AFNI [version AFNI_2008_07_18_1710), that follows the original method described by Glover et al. (2000)]. The input data were raw fMRI data with only volume registration performed. Since respiration could not be monitored during the acquisitions due to technical limitations, breathing noise was estimated with PESTICA [Physiologic EStimation by Temporal ICA (Beall and Lowe, 2007)] from the data themselves. The respiration timecourses obtained in this way were then included in RETROICOR correction as well.

For two patients (Table 1, subjects 2 and 5) the disease symptoms were on the left at onset, corresponding to the right brain hemisphere, so we flipped MR functional and structural images in the right/left direction, in order to have all patients’ predominantly affected hemisphere on the left side of the brain. Structural images were then registered to a symmetrical version of MNI template created by copying, flipping along the x axis and averaging the original and the mirrored version of the template, similarly to the procedure used by Kwak et al. (2012).

**Table 1:** Demographic and clinical data of the patients at the time of the MR session.

N	1	2	3	4	5	6	Mean	(*SD*)
Age (yrs)	46	58	52	46	48	57	51.2	(5.4)
Sex	F	M	F	M	M	M	–	–
Age at onset (yrs)	60	55	48	41	54	51	51.5	(6.5)
Side of onset	R	L	R	R	L	R	–	–
Disease duration (yrs)	6	3	4	5	4	6	4.7	(1.2)
HY stage	2	2	2	2	2	2	2	–
UPDRS III - OFF	19	17	18	17	19	15	17.5	(1.5)
UPDRS III - ON	9	6	10	8	10	10	8.8	(1.6)
UPDRS III reduction (%)	53	65	44	53	48	33	49.3^∗^	(10.7)

*Yrs, years; HY, Hoehn and Yahr staging of severity of Parkinson disease; UPDRS, unified Parkinson’s Disease Rating Scale reported as score; ^∗^significant (*p* < 0.05).*

Functional MR data preprocessing also included high-pass filtering (cut-off = 100 s), motion correction (motion parameters were then added as confounding variables to the model), slice timing correction, brain extraction and spatial smoothing (Gaussian filter, FWHM = 6 mm). Functional data were aligned to structural images using a linear registration (flirt) and structural images were non-linearly aligned to standard MNI space (fnirt), so that, combining the two steps, functional images could be registered to MNI as well. Once ROIs were aligned to fMRI space, their voxel time series were averaged. The first-level of the analysis was based on a general linear model, constructed with the time series of each seed used separately as a regressor. Clusters were determined in Z-statistic images (|*Z*|≥ 2.3) and the family wise-error was controlled with a cluster significance threshold of *p* = 0.05. A second level within-subject fixed-effects analysis was then performed, in order to combine the results of the two OFF resting state runs and the two ON runs, giving each subject’s mean response for each of the two conditions. Finally, the third-level analysis was a mixed-effects group statistics: a two-sample paired *t*-test was performed to compare the connectivity with each seed before and after L-dopa. Clusters were determined with a threshold of *Z* = 2.3 then family wise-error was controlled with a cluster significance threshold of *p* = 0.05. We did not explore whether the variations of brain connectivity correlated with the disease severity because of the small cohort and the homogeneity of the UPDRS motor score in the OFF state.

## Results

Two out of eight patients were unable to complete the EEG-fMRI acquisition protocol. Demographic and clinical data are reported in Table 1.

Patients’ motor performances as assessed by the UPDRS were significantly ameliorated when on medication than when off medication (UPDRS score reduction after L-dopa: 49%, *p* < 0.05).

### fMRI

**FIGURE 1:**
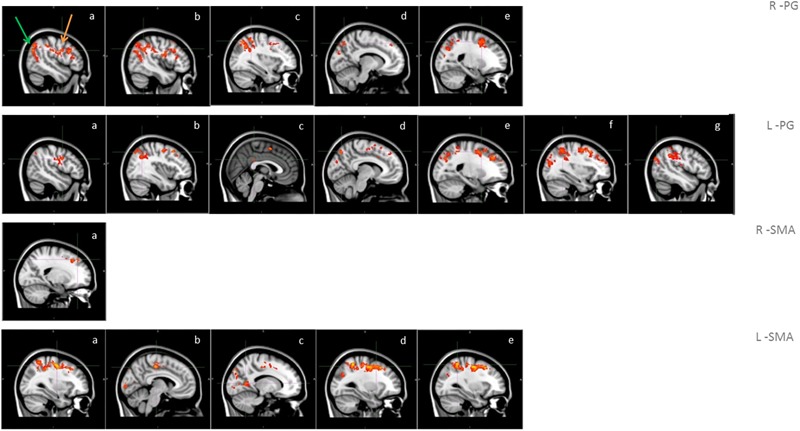
Functional connectivity in the group analysis of ON state vs. OFF state; images show sagittal views of the main areas with significant clusters of voxels, superimposed on the MNI template; box R-PG: connectivity with seed in right precentral gyrus (a: occipital cortex -green arrow-, motor areas -orange arrow-, b: angular gyrus, c: superior parietal lobe, d: precuneus, e: superior frontal gyrus); box L-PG: connectivity with seed in left precentral gyrus (a: motor areas, b: superior frontal gyrus, c: superior parietal lobe, d: angular gyrus, e: precuneus, f: cingulum, g: occipital cortex); box R-SMA: connectivity with seed in right SMA (a: frontal pole – superior frontal gyrus); box L-SMA: connectivity with seed in left SMA (a,b: motor areas, c: precuneus, d: middle/superior frontal gyrus, e: superior parietal lobe).

A significantly higher connectivity with all four seeds was found for the contrast ON vs. OFF (Figure 1 and Table 2). Brain areas showing increased connectivity with both L-PG and R-PG after L-dopa intake were right angular gyrus, middle cingulate gyrus, posterior cingulate gyrus, bilateral middle frontal gyrus, superior frontal gyrus, superior occipital cortex, superior parietal gyrus, postcentral gyrus, precentral gyrus, precuneus and SMA, while left middle and posterior cingulate gyri showed increased connectivity only with L-PG.

**Table 2:** Brain areas showing a significant variation in fMRI connectivity with the four seeds (R-PG, L-PG, R-SMA, and L-SMA) when comparing the ON and the OFF state.

BRAIN AREAS	BRODMANN AREAS	R-PG		L-PG		R-SMA		L-SMA	
		VOXELS	Z MAX	VOXELS	Z MAX	VOXELS	Z MAX	VOXELS	Z MAX
Angular gyrus L	39, 19, 22	72	2.83	33	2.86	0	0	0	0
Angular gyrus R	39, 19, 22	685	3.29	195	2.97	0	0	51	2.80
Middle Cingulate L	31, 32, 23, 24	0	0.00	7	2.50	0	0	78	3.19
Middle Cingulate R	31, 32, 23, 24	13	2.57	3	2.46	0	0	31	2.60
Posterior Cingulate L	31, 23, 118	0	0.00	4	2.74	0	0	0	0
Posterior Cingulate R	31, 23, 118	40	3.01	23	2.91	0	0	5	2.63
Middle Frontal L	6, 8, 9, 10	259	2.88	696	3.29	0	0	469	3.36
Middle Frontal R	6, 8, 9, 10	211	3.04	361	3.31	363	3.09	398	3.32
Superior Frontal L	6, 8, 9, 10	132	2.97	413	3.20	0	0	196	3.12
Superior Frontal R	6, 8, 9, 10	257	2.91	356	3.05	158	2.99	233	3.24
Superior Occipital L	18, 19, 7, 31	187	2.98	130	3.04	0	0	144	3.07
Superior Occipital R	18, 19, 7, 31	53	2.81	39	2.91	0	0	0	0
Superior Parietal L	7, 19	167	2.91	207	2.90	0	0	162	3.35
Superior Parietal R	7, 19	115	2.95	17	2.77	0	0	191	3.12
Postcentral gyrus L	1, 2, 3, 40, 43	201	2.90	235	2.86	0	0	373	3.23
Postcentral gyrus R	1, 2, 3, 40, 43	121	2.93	134	3.11	0	0	164	3.19
Precentral gyrus L	3, 4, 6	15	2.54	154	2.99	0	0	370	3.38
Precentral gyrus R	3, 4, 6	61	3.05	264	3.08	11	2.61	250	3.46
Precuneus L	31, 7, 23, 29	57	2.79	59	2.96	0	0	8	2.46
Precuneus R	31, 7, 23, 29	141	2.85	6	2.35	0	0	62	2.81
SMA L	6	18	2.81	150	3.30	0	0	57	2.88
SMA R	6	1	2.33	58	3.25	0	0	97	2.84

*L, left; R, right; SMA, supplementary motor area; PG, precentral gyrus.*

As for the SMA, L-SMA showed increased connectivity with right angular gyrus, posterior cingulate gyrus, left superior occipital cortex, bilateral middle cingulate gyrus, middle and superior frontal gyri, superior parietal gyrus, postcentral gyrus, precentral gyrus, precuneus and SMA, while R-SMA had an increased connectivity only with right middle and superior frontal giri and right precentral gyrus. The group analysis showed no significant variations in connectivity for the contrast OFF vs. ON.

### EEG

When evaluating the connections among the four seeds of the motor system (R-PG, L-PG, R-SMA, and L-SMA) we did not find any significant difference in PDC in ON vs. OFF (*p* > 0.05) for all the studied frequencies (alpha, beta, and theta). Although not significant, a trend toward a decreased FC in ON vs. OFF among the different structures of the motor system was observed for each frequency.

Similarly, no significant differences for ON vs. OFF were observed when evaluating whether the motor system was changing its outflow (in term of summed outflow from the four ROIs) toward the remaining regions of the brain. In this case as well, we can observe a tendency toward a decreased summed outflow from the motor system, especially concerning the alpha and the theta bands, after L-dopa intake.

As for the PDC from each ROI of the whole brain, we observed a strong tendency (*p* < 0.06) toward a change in the summed outflow between ON and OFF conditions mainly in the following structures: posterior cingulate, left amygdala, left hippocampus, right anterior cingulate and right lingual gyrus in the alpha band; left amygdala, bilateral hippocampi and right anterior cingulate in the beta band; posterior cingulate, left amygdala, bilateral hippocampi and right anterior cingulate in the theta band (Supplementary Figures 1–3).

**FIGURE 2:**
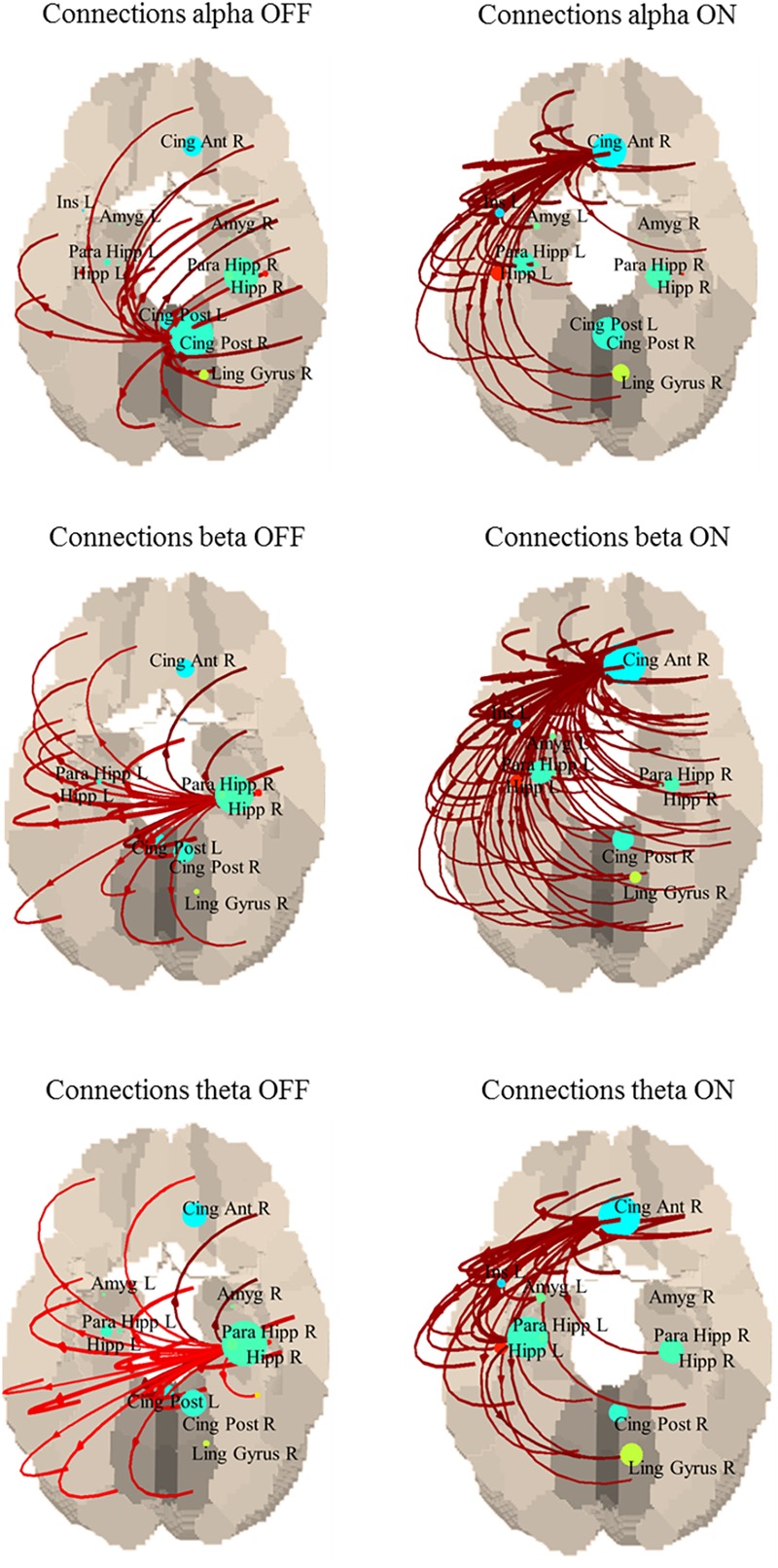
Connections between each ROI in OFF (left) and ON (right). Only the strongest 30% connections are shown. Alpha band: the main driver of connections is the posterior cingulate in the OFF state and the anterior cingulate in the ON state. Beta band: the main driver of connections is the right hippocampus in the OFF state and the anterior cingulate in the ON state. Theta band: the main driver of connections is the right hippocampus in the OFF state and the anterior cingulate in the ON state.

Although this difference was not statistically significant, an interesting common behavior is that in the OFF state, the strongest connections originated from the posterior regions of the default mode network (DMN), while in ON they came from the anterior cingulate, part of the anterior portion of the DMN (Figure 2). Specifically, in the alpha band the main driver of connection is the posterior cingulate in the OFF state and the anterior cingulate in the ON state; in the beta band the main driver of connections is the right hippocampus in the OFF state and the anterior cingulate in the ON state; in the theta band the main driver of connections is the right hippocampus in the OFF state and the anterior cingulate in the ON state.

## Discussion

This exploratory pilot study is the first that describes the potentialities of resting state functional connectivity measured simultaneously with EEG and fMRI in early-stage PD patients to describe acute L-dopa effect.

Resting state fMRI results showed, after the administration of L-dopa, an increased bilateral connectivity of PG and SMA with cortical areas that belong to three resting state brain networks: sensorimotor, DMN and dorsal-attention network (Figure 1 and Table 2). The sensorimotor network deals with the integration of sensitive and motor stimuli and its characteristic spatial pattern includes pre- and post-central gyri, extending from the superior bank of the sylvian fissure to the medial wall of the interhemispheric fissure and the SMA (Damoiseaux et al., 2006).

Our findings of increased connectivity within the sensorimotor system after the intake of L-dopa are in line with previous results (Esposito et al., 2013) that showed enhanced function connectivity within the SMA in drug-naïve PD patients after acute L-dopa administration. Effects of L-dopa administration on resting connectivity were also investigated in patients with advanced PD, and a higher resting state functional connectivity was found between striatum and prefrontal cortex, and a lower connectivity between pallidum, STN, and supplementary and primary motor cortices (Akram et al., 2017). Compared to previous studies (Wu et al., 2012; Esposito et al., 2013; Akram et al., 2017) we detected limited variations of functional connectivity between the motor cortex and deep brain structures: this result may be related to differences in clinical samples and methodological approaches.

The DMN is typically constituted by ventro-medial prefrontal cortex, cingulate cortex, inferior parietal lobule, lateral temporal cortex, dorsal medial prefrontal cortex and hippocampus, and it is particularly active during rest condition while it deactivates when specific goal-directed behavior is needed (Damoiseaux et al., 2006). For PD patients, a stronger connection of DMN with PG and SMA after the administration of L-dopa may contribute to an increased readiness to plan and perform movements. In line with this hypothesis, Krajcovicova et al. (2012) found no differences in DMN integrity between PD patients on dopaminergic medication and healthy controls, suggesting that dopaminergic therapy may have specific effects on restoring default mode functional integrity. It has also been shown (Van Eimeren et al., 2009) that in PD patients, deficits in executive tasks such as planning and set-shifting were associated with less deactivation of posterior cingulate cortex and precuneus. On the other hand, a resting state fMRI study in cognitively unimpaired PD patients (Tessitore et al., 2012b) showed lower DMN functional connectivity in patients compared to healthy controls, and this impairment was uncorrelated with the L-dopa dosage.

The dorsal attention system is crucial in voluntary orientation and attention and it mainly involves the intraparietal sulcus, the frontal eye field, the junction of precentral and superior frontal sulci, and middle and superior frontal gyri (Damoiseaux et al., 2006). In our study, structures of attentive and executive systems showed increased functional connectivity with cortical motor areas after L-dopa administration. This may suggest that dopaminergic medication could be of help to PD patients as for their executive dysfunction, by improving the cognitive, attentional and executive steps, essential for movements preparation. When activations and connectivity were investigated during the performance of a motor-attentional task (Rowe et al., 2002), an attentional modulation of connectivity was observed only in the healthy subjects, but not in PD patients. In particular, the attention to action led to further activations of prefrontal, parietal, and para-cingulate cortices and SMA. The engagement of the attentional control network was also investigated in PD and a lower activation of frontal and parietal hubs of the dorsal attention network was described (Shine et al., 2014). Reduced functional connectivity has been reported within both the executive-attention and visual networks in PD patients with freezing compared to those without (Tessitore et al., 2012a), suggesting that freezing may be related to a dysfunction of the visuo-spatial network. As none of our early-stage PD patients presented freezing, the freezing related network may be impaired before clinical manifestation.

Partial directed coherence analysis of EEG data acquired during the fMRI scans revealed no statistically significant motor network changes when comparing ON vs. OFF. Nevertheless, the results suggest an interesting tendency toward a decreased coherence after the intake of L-dopa within the motor system and between the motor system and the rest of the brain. However, the effect of L-dopa on cortico-cortical coherence is still debated and opposing results can be found in the literature. Previous resting state scalp EEG studies (Silberstein et al., 2005; Hirschmann et al., 2013) have shown that STN-cortical and cortico-cortical coherence correlate with the severity of parkinsonism and that this coupling was modulated by L-dopa therapy and STN stimulation. When MEG and subthalamic local field potential recordings were used to investigate connectivity in PD patients (Litvak et al., 2011), two major spatio-temporal patterns of coupling between cortex and STN were observed, in alpha and beta bands. In the alpha band, coherence between STN and bilateral temporo-parietal cortex and brainstem has been described, suggesting a possible attentional role; in the beta band the pattern involved the STN and the ipsilateral anterior parietal and frontal cortices, suggesting an involvement in the executive functions. In this study, dopaminergic medication increased beta coherence between STN and prefrontal cortex. However, later studies did not confirm this effect of pharmacological intervention on cortical coherence. For example, the evaluation of the direct transfer function between STN and cortex at rest and during movement (Lalo et al., 2008), with and without pharmacological dopaminergic input, showed that the beta band coherence did not change after dopaminergic therapy. MEG coherence among SMA and other motor areas has also been investigated during resting and during isometric muscular contraction (Pollok et al., 2013), before and after L-dopa intake. Interestingly, an increased SMA–M1 coherence in OFF during isometric contraction was found, and it was remedied by L-dopa. Nevertheless, coherence strength for the resting state did not differ after L-dopa intake, suggesting that SMA–M1 coherence might be more related to movement execution than to the rest condition. Besides, a high-density EEG study investigated the effect of acute L-dopa administration on movement-related cortical oscillations, showing that L-dopa reduce the exaggerated movement-related beta-band desynchronization in the SMA that was observed in PD patients compared to healthy subjects (Chung et al., 2018) and that functional brain activity in the basal ganglia pathways relate to the response of beta-band cortical activity to levodopa.

When whole brain outflow was considered, the regions that showed a stronger tendency to change their connectivity are the posterior and anterior cingulate, and the right hippocampus, mainly ascribable to the DMN. This is quite interesting as changes in the connectivity with the DMN were also observed in our fMRI results, and we discussed above the crucial role of this network and its connections with the sensorimotor areas in PD patients. Moreover, there seems to be a shift of the involvement in connections toward the anterior subpart of DMN after the intake of L-dopa. Structural and functional alterations of cingulate cortex has been previously shown in PD patients. For example, increased functional connectivity and higher eigenvector centrality (a network measure that allow to identify prominent nodes in the whole brain network) in the posterior cingulate gyrus and lower centrality for the anterior cingulate gyrus (de Schipper et al., 2018), white matter microstructural alterations in the cingulate bundle near the orbital and anterior cingulate gyrus (Albrecht et al., 2019), or a loss of integrity in cingulate structural covariance network, for both anterior and posterior cingulate cortices (de Schipper et al., 2017). It would be suggestive to hypothesize that what we see is a relative normalization of the connectivity pattern, speculating about different roles of anterior and posterior cingulate cortices, but it might be a rash speculation, as a group of healthy controls is not available and there are no similar findings in literature to compare with.

The absence of clear PDC findings in the present study may be related to methodological limitations. Simultaneous recordings to MRI induces artifacts in the EEG, reducing the quality of the trace. The gradient artifact is the most important one, with an amplitude of about 50 times the background EEG. The most widely used method to remove it consists of estimating the artifact and subtracting it from each frame (Bénar et al., 2003). In our case, the frequency removed by the gradient artifact correction was within the beta band. Furthermore, when removing the cardioballistic artifact by ICA, there is also a risk of removing components of signal which are actually coming from the brain. A precise description of the influence of MRI gradients and ECG artifacts on multivariate measures performed on EEG signals, such as PDC, is still lacking. Further exploration of these effects by using various acquisition protocols and scanning equipment are necessary to properly evaluate the method’s sensitivity to these artifacts.

The main limit of this exploratory study is the small number of recruited patients. This poses substantial limitation to the generalizability of the results, indeed the present study should be considered a pilot investigation, that might show the potentialities of EEG and fMRI combination in PD patients, but without any presumptions of fully reliable and solid conclusions. Further studies in larger samples are needed to confirm and expand the present results. However, the sample is very homogeneous as all PD patients are in an early stage of the disease course with comparable motor impairment. Despite the small cohort, both fMRI and EEG findings are directionally similar, showing increased connectivity after L-dopa intake in PD patients, particularly for motor areas and their connections with dorsal attention and DMN areas. Taking into consideration the novelty of this approach in PD patients, this pilot study showed the potentialities of this methodology to better understand the mechanisms underlying electrical and hemodynamic functional connectivity changes in these patients.

## Data Availability

The datasets generated for this study are available on request to the corresponding author.

## Ethics Statement

This study was carried out in accordance with the Declaration of Helsinki. The protocol was approved by the Local Public Health Service (AUSL) of Bologna Ethics Committee and all participants provided written informed consent.

## Author Contributions

FP, ClT, GR, PC, GC-B, CaT, RL, and PT contributed to the conception of the study. SE, FP, ClT, GR, LG, LF, FB, CB, DM, and CaT contributed to the acquisitions. SE, FP, ClT, LF, AC, and LT contributed to the data analysis. SE, FP, ClT, GR, LF, AC, PC, GC, CaT, RL, and PT contributed to the results interpretation. SE, FP, and ClT contributed to manuscript preparation. All authors contributed to the manuscript revision and approved the final version of the manuscript.

## Conflict of Interest Statement

The authors declare that the research was conducted in the absence of any commercial or financial relationships that could be construed as a potential conflict of interest.

## Footnotes

1 https://sites.google.com/site/cartoolcommunity/home

## References

[B1] Akram H. Wu C. Hyam J. Foltynie T. Limousin P. De Vita E. (2017). l-Dopa responsiveness is associated with distinctive connectivity patterns in advanced Parkinson’s disease. *Mov. Disord.* 32 874–883. 10.1002/mds.27017 28597560 PMC7116734

[B2] Albrecht F. Ballarini T. Neumann J. Schroeter M. L. (2019). FDG-PET hypometabolism is more sensitive than MRI atrophy in Parkinson’s disease: a whole-brain multimodal imaging meta-analysis. *Neuroimage Clin.* 21:101594. 10.1016/j.nicl.2018.11.004 30514656 PMC6413303

[B3] Allen P. J. Josephs O. Turner R. (2000). A method for removing imaging artifact from continuous EEG recorded during functional MRI. *NeuroImage* 12 230–239. 10.1006/nimg.2000.0599 10913328

[B4] Astolfi L. Cincotti F. Mattia D. De Vico Fallani F. Tocci A. Colosimo A. (2008). Tracking the time-varying cortical connectivity patterns by adaptive multivariate estimators. *IEEE Trans. Biomed. Eng.* 55 902–913. 10.1109/TBME.2007.905419 18334381

[B5] Bajaj S. Krismer F. Palma J. A. Wenning G. K. Kaufmann H. Poewe W. (2017). Diffusion-weighted MRI distinguishes Parkinson disease from the parkinsonian variant of multiple system atrophy: a systematic review and meta-analysis. *PLoS One* 12:e0189897. 10.1371/journal.pone.0189897 29287113 PMC5747439

[B6] Beall E. B. Lowe M. J. (2007). Isolating physiologic noise sources with independently determined spatial measures. *NeuroImage* 37 1286–1300. 10.1016/j.neuroimage.2007.07.004 17689982

[B7] Bénar C. Aghakhani Y. Wang Y. Izenberg A. Al-Asmi A. Dubeau F. (2003). Quality of EEG in simultaneous EEG-fMRI for epilepsy. *Clin. Neurophysiol.* 114 569–580. 10.1016/S1388-2457(02)00383-8 12705438

[B8] Birot G. Spinelli L. Vulliémoz S. Mégevand P. Brunet D. Seeck M. (2014). Head model and electrical source imaging: a study of 38 epileptic patients. *Neuroimage Clin.* 5 77–83. 10.1016/j.nicl.2014.06.005 25003030 PMC4081973

[B9] Boecker R. Holz N. E. Buchmann A. F. Blomeyer D. Plichta M. M. Wolf I. (2014). Impact of early life adversity on reward processing in young adults: EEG-fMRI results from a prospective study over 25 years. *PLoS One* 9:e104185. 10.1371/journal.pone.0104185 25118701 PMC4131910

[B10] Brown P. (2003). Oscillatory nature of human basal ganglia activity: relationship to the pathophysiology of Parkinson’s disease. *Mov. Disord.* 18 357–363. 10.1002/mds.10358 12671940

[B11] Brown P. Marsden C. D. (1998). What do the basal ganglia do? *Lancet* 351 1801–1804. 10.1016/S0140-6736(97)11225-9 9635969

[B12] Brown P. Oliviero A. Mazzone P. Insola A. Tonali P. Di Lazarro V. (2001). Dopamine dependency of oscillations between subthalamic nucleus and pallidum in Parkinson’s disease. *J. Neurosci.* 21 1033–1038. 10.1523/JNEUROSCI.21-03-01033.2001 11157088 PMC6762327

[B13] Brueggen K. Fiala C. Berger C. Ochmann S. Babiloni C. Teipel S. J. (2017). Early changes in alpha band power and DMN BOLD activity in alzheimer’s disease: a simultaneous resting state EEG-fMRI study. *Front. Aging Neurosci.* 6:319 10.3389/fnagi.2017.00319 PMC5635054 29056904

[B14] Buhmann C. Glauche V. Sturenburg H. J. Oechsner M. Weiller C. Buchel C. (2003). Pharmacologically modulated fMRI-cortical responiveness to levodopa in drug-naive hemiparkinsonian patients. *Brain* 126 451–461. 10.1093/brain/awg033 12538411

[B15] Chung J. W. Burciu R. G. Ofori E. Coombes S. A. Christou E. A. Okun M. S. (2018). Beta-band oscillations in the supplementary motor cortex are modulated by levodopa and associated with functional activity in the basal ganglia. *Neuroimage Clin.* 18 559–571. 10.1016/j.nicl.2018.05.021 29984164 PMC6029579

[B16] Coito A. Genetti M. Pittau F. Iannotti G. R. Thomschewski A. Höller Y. (2016). Altered directed functional connectivity in temporal lobe epilepsy in the absence of interictal spikes: a high density EEG study. *Epilepsia* 57 402–411. 10.1111/epi.13308 26890734

[B17] Coito A. Plomp G. Genetti M. Abela E. Wiest R. Seeck M. (2015). Dynamic directed interictal connectivity in left and right temporal lobe epilepsy. *Epilepsia* 56 207–217. 10.1111/epi.12904 25599821

[B18] Damoiseaux J. S. Rombouts S. A. Barkhof F. Scheltens P. Stam C. J. Smith S. M. (2006). Consistent resting-state networks across healthy subjects. *PNAS* 103 13848–13853. 10.1073/pnas.0601417103 16945915 PMC1564249

[B19] de Schipper L. J. Hafkemeijer A. van der Grond J. Marinus J. Henselmans J. M. L. van Hilten J. J. (2018). Altered whole-brain and network-based functional connectivity in parkinson’s disease. *Front. Neurol.* 9:419. 10.3389/fneur.2018.00419 29928255 PMC5997827

[B20] de Schipper L. J. van der Grond J. Marinus J. Henselmans J. M. L. van Hilten J. J. (2017). Loss of integrity and atrophy in cingulate structural covariance networks in Parkinson’s disease. *Neuroimage Clin.* 15 587–593. 10.1016/j.nicl.2017.05.012 28652971 PMC5477092

[B21] Drissi N. M. Szakács A. Witt S. T. Wretman A. Ulander M. Ståhlbrandt H. (2016). Altered brain microstate dynamics in adolescents with narcolepsy. *Front. Hum. Neurosci.* 3:369. 10.3389/fnhum.2016.00369 27536225 PMC4971065

[B22] Esposito F. Tessitore A. Giordano A. De Micco R. Paccone A. Conforti R. (2013). Rhythm-specific modulation of the sensorimotor network in drug-naive patients with Parkinson’s disease by levodopa. *Brain* 136 710–725. 10.1093/brain/awt007 23423673

[B23] Gelb D. J. Oliver E. Gilman S. (1999). Diagnostic criteria for Parkinson disease. *Arch Neurol.* 56 33–39. 9923759 10.1001/archneur.56.1.33

[B24] Glover G. H. Li T. Q. Ress D. (2000). Image-based method for retrospective correction of physiological motion effects in fMRI: RETROICOR. *Magn. Reson. Med.* 44 162–167. 10.1002/1522-2594(200007)44:1<162::aid-mrm23>3.3.co;2-5 10893535

[B25] Grave de Peralta Menendez R. Murray M. M. Michel C. M. Martuzzi R. Gonzalez Andino S. L. (2004). Electrical neuroimaging based on biophysical constraints. *Neuroimage* 21 527–539. 10.1016/j.neuroimage.2003.09.051 14980555

[B26] Haslinger B. Erhard P. Kampfe N. Boecker H. Rummeny E. Schwaiger M. (2001). Event-related functional magnetic resonance imaging in Parkinson’s disease before and after levodopa. *Brain* 124 558–570. 10.1093/brain/124.3.558 11222456

[B27] Heim B. Krismer F. De Marzi R. Seppi K. (2017). Magnetic resonance imaging for the diagnosis of Parkinson’s disease. *J. Neural Transm.* 124 915–964. 10.1007/s00702-017-1717-8 28378231 PMC5514207

[B28] Hirschmann J. Özkurt T. E. Butz M. Homburger M. Elben S. Hartmann C. J. (2011). Distinct oscillatory STN-cortical loops revealed by simultaneous MEG and local field potential recordings in patients with Parkinson’s disease. *NeuroImage* 55 1159–1168. 10.1016/j.neuroimage.2010.11.063 21122819

[B29] Hirschmann J. Özkurt T. E. Butz M. Homburger M. Elben S. Hartmann C. J. (2013). Differential modulation of STN-cortical and cortico-muscular coherence by movement and levodopa in Parkinson’s disease. *NeuroImage* 68 203–213. 10.1016/j.neuroimage.2012.11.036 23247184

[B30] Hoehn M. M. Yahr M. D. (1967). Parkinsonism: onset, progression and mortality. *Neurology* 17 427–442. 6067254 10.1212/wnl.17.5.427

[B31] Huster R. J. Debener S. Eichele T. Herrmann C. S. (2012). Methods for simultaneous EEG-fMRI: an introductory review. *J. Neurosci.* 32 6053–6060. 10.1523/JNEUROSCI.0447-12.2012 22553012 PMC6622140

[B32] Jankovic J. (2008). Parkinson’s disease: clinical features and diagnosis. *J. Neurol. Neurosurg. Psychiatry* 4 368–376. 10.1136/jnnp.2007.131045 18344392

[B33] Jenkinson M. Beckmann C. F. Behrens T. E. Woolrich M. W. Smith S. M. (2012). FSL. *NeuroImage* 62 782–790. 10.1016/j.neuroimage.2011.09.015 21979382

[B34] Krajcovicova L. Mikl M. Marecek R. Rektorova I. (2012). The default mode network integrity in patients with Parkinson’s disease is levodopa equivalent dose-dependent. *J. Neural Transm.* 119 443–454. 10.1007/s00702-011-0723-5 22002597

[B35] Kwak Y. Peltier S. J. Bohnen N. I. Müller M. L. Dayalu P. Seidler R. D. (2012). L-DOPA changes spontaneous low-frequency BOLD signal oscillations in Parkinson’s disease: a resting state fMRI study. *Front. Syst. Neurosci.* 6:52. 10.3389/fnsys.2012.00052 22783172 PMC3389385

[B36] Lalo E. Thobois S. Sharott A. Polo G. Mertens P. Pogosyan A. (2008). Patterns of bidirectional communication between cortex and basal ganglia during movement in patients with Parkinson disease. *J. Neurosci.* 28 3008–3016. 10.1523/JNEUROSCI.5295-07.2008 18354004 PMC6670699

[B37] Lei X. Wang Y. Yuan H. Mantini D. (2014). Neuronal oscillations and functional interactions between resting state networks. *Hum. Brain Mapp.* 35 3517–3528. 10.1002/hbm.22418 25050432 PMC6869195

[B38] Levy R. Ashby P. Hutchison W. D. Lang A. E. Lozano A. M. Dostrovsky J. O. (2002a). Dependence of subthalamic nucleus oscillations on movement and dopamine in Parkinson’s disease. *Brain* 125 1196–1209. 10.1093/brain/awf128 12023310

[B39] Levy R. Hutchison W. D. Lozano A. M. Dostrovsky J. O. (2002b). Synchronized neuronal discharge in the basal ganglia of parkinsonian patients is limited to oscillatory activity. *J. Neurosci.* 22 2855–2861. 10.1523/JNEUROSCI.22-07-02855.2002 11923450 PMC6758317

[B40] Litvak V. Jha A. Eusebio A. Oostenveld R. Foltynie T. Limousin P. (2011). Resting oscillatory corticosubthalamic connectivity in patients with Parkinson’s disease. *Brain* 134 359–374. 10.1093/brain/awq332 21147836

[B41] Marsden C. D. Obeso J. A. (1994). The functions of the basal ganglia and the paradox of stereotaxic surgery in Parkinson’s disease. *Brain* 117 877–897. 10.1093/brain/117.4.877 7922472

[B42] McPherson A. (1970). Convulsive seizures and electroencephalogram changes in three patients during levodopa therapy. *Neurology* 20 41–45. 10.1212/WNL.20.12_Part_2.41 5531293

[B43] Morisi R. Manners D. N. Gnecco G. Lanconelli N. Testa C. Evangelisti S. (2018). Multi-class parkinsonian disorders classification with quantitative MR markers and graph-based features using support vector machines. *Parkinson. Relat. Disord.* 47 64–70. 10.1016/j.parkreldis.2017.11.343 29208345

[B44] Neufeld M. Y. Inzelberg R. Korczyn A. D. (1988). EEG in demented and non-demented parkinsonian patients. *Acta Neurol Scand.* 78 1–5. 10.1111/j.1600-0404.1988.tb03609.x 3176875

[B45] Obeso J. A. Rodriguez M. C. DeLong M. R. (1997). Basal ganglia pathophysiology. a critical review. *Adv. Neurol.* 74 3–18. 9348398

[B46] Ohara S. Mima T. Baba K. Ikeda A. Kunieda T. Matsumoto R. (2001). Increased synchronization of cortical oscillatory activities between human supplementary motor and primary sensorimotor areas during voluntary movements. *J. Neurosci.* 21 9377–9393. 10.1523/JNEUROSCI.21-23-09377.2001 11717371 PMC6763917

[B47] Plomp G. Quairiaux C. Michel C. M. Astolfi L. (2014). The physiological plausibility of time-varying Granger-causal modeling: normalization and weighting by spectral power. *Neuroimage* 97 206–216. 10.1016/j.neuroimage.2014.04.016 24736179

[B48] Pollok B. Kamp D. Butz M. Wojtecki L. Timmermann L. Südmeyer M. (2013). Increased SMA-M1 coherence in Parkinson’s disease – Pathophysiology or compensation? *Exp. Neurol.* 247 178–181. 10.1016/j.expneurol.2013.04.013 23664959

[B49] Priori A. Foffani G. Pesenti A. Bianchi A. Chiesa V. Baselli G. (2002). Movement-related modulation of neural activity in human basal ganglia and its L-DOPA dependency: recordings from deep brain stimulation electrodes in patients with Parkinson’s disease. *Neurol Sci.* 23(Suppl. 2) S101–S102. 10.1007/s100720200089 12548363

[B50] Rizzo G. Tonon C. Lodi R. (2012). Looking into the brain: How can conventional, morphometric and functional MRI help in diagnosing and understanding PD? *Basal Ganglia* 2 175–182. 10.1016/j.baga.2012.06.001

[B51] Rosenkranz K. Lemieux L. (2010). Present and future of simultaneous EEG-fMRI. *MAGMA* 23 309–316. 10.1007/s10334-009-0196-9 20101434

[B52] Rowe J. Stephan K. E. Friston K. Frackowiak R. Lees A. Passingham R. (2002). Attention to action in Parkinson’s disease. Impaired effective connectivity among frontal cortical regions. *Brain* 125 276–289. 10.1093/brain/awf036 11844728

[B53] Schneider S. Wagels L. Haeussinger F. B. Fallgatter A. J. Ehlis A. C. Rapp A. M. (2015). Haemodynamic and electrophysiological markers of pragmatic language comprehension in schizophrenia. *World J. Biol. Psychiatry* 16 398–410. 10.3109/15622975.2015.1019359 25816925

[B54] Serrien D. J. Brown P. (2003). The integration of cortical and behavioural dynamics during initial learning of a motor task. *Eur. J. Neurosci.* 17 1098–1104. 10.1046/j.1460-9568.2003.02534.x 12653986

[B55] Sharman M. Valabregue R. Perlbarg V. Marrakchi-Kacem L. Vidailhet M. Benali H. (2013). Parkinson’s disease patients show reduced cortical-subcortical sensorimotor connectivity. *Mov. Disord.* 28 447–454. 10.1002/mds.25255 23144002

[B56] Shine J. M. Halliday G. M. Gilat M. Matar E. Bolitho S. J. Carlos M. (2014). The role of dysfunctional attentional control networks in visual misperceptions in parkinson’s disease. *Hum. Brain Mapp.* 35 2206–2219. 10.1002/hbm.22321 23760982 PMC6869072

[B57] Silberstein P. Oliviero A. Di Lazzaro V. Insola A. Mazzone P. Brown P. (2005). Oscillatory pallidal local field potential activity inversely correlates with limb dyskinesias in Parkinson’s disease. *Exp. Neurol.* 194 523–529. 10.1016/j.expneurol.2005.03.014 16022875

[B58] Syed Nasser N. Ibrahim B. Sharifat H. Abdul Rashid A. Suppiah S. (2019). Incremental benefits of EEG informed fMRI in the study of disorders related to meso-corticolimbic dopamine pathway dysfunction: a systematic review of recent literature. *J. Clin. Neurosci.* 10.1016/j.jocn.2019.03.054 [Epub ahead of print]. 30955950

[B59] Tagliazucchi E. Laufs H. (2014). Decoding wakefulness levels from typical fMRI resting-state data reveals reliable drifts between wakefulness and sleep. *Neuron* 82 695–708. 10.1016/j.neuron.2014.03.020 24811386

[B60] Tessitore A. Amboni M. Esposito F. Russo A. Picillo M. Marcuccio L. (2012a). Resting state brain connectivity in patients with Parkinson’s disease and freezing of gait. *Parkinson. Relat. Disord.* 18 781–787. 10.1016/j.parkreldis.2012.03.018 22510204

[B61] Tessitore A. Esposito F. Vitale C. Santangelo G. Amboni M. Russo A. (2012b). Default-mode network connectivity in cognitively unimpaired patients with Parkinson disease. *Neurology* 79 2226–2232. 10.1212/WNL.0b013e31827689d6 23100395

[B62] Van Eimeren T. Monchi O. Ballanger B. Strafella A. P. (2009). Dysfunction of the default mode network in parkinson disease. *Arch. Neurol.* 66 877–883. 10.1001/archneurol.2009.97 19597090 PMC2972248

[B63] Van Mierlo P. Carrette E. Hallez H. Vonck K. Van Roost D. Boon P. (2011). Accurate epileptogenic focus localization through time-variant functional connectivity analysis of intracranial electroencephalographic signals. *Neuroimage* 56 1122–1133. 10.1016/j.neuroimage.2011.02.009 21316472

[B64] Vitali P. Di Perri C. Vaudano A. E. Meletti S. Villani F. (2015). Integration of multimodal neuroimaging methods: a rationale for clinical applications of simultaneous EEG-fMRI. *Funct. Neurol.* 30 9–20. 26214023 PMC4520679

[B65] Williams D. Tijssen M. Van Bruggen G. Bosch A. Insola A. Di Lazzaro V. (2002). Dopamine-dependent changes in the functional connectivity between basal ganglia and cerebral cortex in humans. *Brain* 125 1558–1569. 10.1093/brain/awf156 12077005

[B66] Wu T. Hallett M. (2005). A functional MRI study of automatic movements in patients with Parkinson’s disease. *Brain* 128 2250–2259. 10.1093/brain/awh569 15958505

[B67] Wu T. Long X. Wang L. Hallett M. Zang Y. Li K. (2011). Functional connectivity of cortical motor areas in the resting state in parkinson’s disease. *Hum. Brain Mapp.* 32 1443–1457. 10.1002/hbm.21118 20740649 PMC6870250

[B68] Wu T. Wang J. Wang C. Hallett M. Zang Y. Wu X. (2012). Basal ganglia circuits changes in Parkinson’s disease patients. *Neurosci. Lett.* 524:55. 10.1016/j.neulet.2012.07.012 22813979 PMC4163196

[B69] Wu T. Wang L. Chena Y. Zhaob C. Li K. Chana P. (2009). Changes of functional connectivity of the motor network in the resting state in Parkinson’s disease. *NeuroImage* 460 6–10. 10.1016/j.neulet.2009.05.046 19463891

[B70] Yeager C. L. Alberts W. Delattre L. D. (1966). Effect of stereotaxic surgery upon electroencephalographic status of parkinsonian patients. *Neurology* 16 904–910.

[B71] Zanigni S. Evangelisti S. Testa C. Manners D. N. Calandra-Buonaura G. Guarino M. (2017). White matter and cortical changes in atypical parkinsonisms: a multimodal quantitative MR study. *Parkinson. Relat. Disord.* 39 44–51. 10.1016/j.parkreldis.2017.03.001 28291592

