# Supplementary material for: L-Dopa Modulation of Brain Connectivity in Parkinson’s Disease Patients: A Pilot EEG-fMRI Study
Source: Front Neurosci. 2019 Jun 14;13:611. doi: 10.3389/fnins.2019.00611 (PMC6587436; doi:10.3389/fnins.2019.00611)
Supplement: Supplementary file 1 [file Data_Sheet_1.docx]

**Supplementary Figure 1.** On the left: outflow for each ROI in the alpha band, in OFF. On the right: same representation in ON. Regions showing a strong tendency (p<0.06) to change their outflow in ON vs. OFF are: posterior cingulate, left amygdala and hippocampus, right anterior cingulate, right lingual gyrus.

**Supplementary Figure 2.** On the left: outflow for each ROI in the beta band, in OFF. On the right: same representation in ON. Regions showing a strong tendency (p<0.06) to change their outflow in ON vs. OFF are: left amygdale, bilateral hippocampi, and right anterior cingulate.

**Supplementary Figure 3.** On the left: outflow for each ROI in the theta band, in OFF. On the right: same representation in ON. Regions showing a strong tendency (p<0.06) to change their outflow in ON vs. OFF are: posterior cingulate, left amygdale, bilateral hippocampi, and right anterior cingulate.
